# Sulforaphane-cysteine elicits apoptosis through JNK-mediated caspase activation in oral squamous cell carcinoma cells

**DOI:** 10.7150/jca.126381

**Published:** 2026-01-01

**Authors:** Yen-Lin Chen, Yi-Tzu Chen, Wei-En Yang, Chun-Wen Su, Meng-Ying Tsai, Shih-Chi Su, Shun-Fa Yang, Chiao-Wen Lin

**Affiliations:** 1 School of Dentistry, Chung Shan Medical University, Taichung, Taiwan.; 2 Department of Dentistry, Chung Shan Medical University Hospital, Taichung, Taiwan.; 3 Institute of Medicine, Chung Shan Medical University, Taichung, Taiwan.; 4 Department of Medical Research, Chung Shan Medical University Hospital, Taichung, Taiwan.; 5 Whole-Genome Research Core Laboratory of Human Diseases, Chang Gung Memorial Hospital, Keelung, Taiwan.; 6 Department of Medical Biotechnology and Laboratory Science, College of Medicine, Chang Gung University, Taoyuan, Taiwan.; 7 Institute of Oral Sciences, Chung Shan Medical University, Taichung, Taiwan.

**Keywords:** oral squamous cell carcinoma, sulforaphane-cysteine, apoptosis, caspase, JNK

## Abstract

Sulforaphane-cysteine (SFN-Cys) is a naturally-occurring form of plant-derived isothiocyanate metabolites that displays several tumor-suppressive properties. However, the oncostatic potential of SFN-Cys on oral squamous cell carcinoma (OSCC) is mostly elusive. In this study, we tried to test whether SFN-Cys affects OSCC to progress and further explored the underlying array of molecular cues that SFN-Cys mediates. Our results demonstrate that SFN-Cys was an effective inducer of cytotoxicity to OSCC cells, accompanied with blockage of cell cycling and promotion of apoptotic events. Moreover, treatment of OSCC cells with SFN-Cys attuned an apoptosis-associated protein regulatory program, underlined by downregulation of apoptosis suppressors (cIAP-1 and XIAP) and activation of caspase cascades. Furthermore, caspase activations in SFN-Cys-treated OSCC cells were affected by the pre-incubation with a specific c-Jun N-terminal kinase (JNK) inhibitor, suggesting a functional link of JNK pathway to SFN-Cys's actions in OSCC cells. Collectively, our data revealed that SFN-Cys hampered cell cycle progression and elicited apoptotic responses in OSCC via a JNK-mediated activation of caspase pathways. These findings provide possible avenues for the application of a natural compound in the management of oral malignancies.

## Introduction

Oral squamous cell carcinoma (OSCC), representing the most prevalent form of oral neoplasm (nearly 90% of all cases), is a common malignant disease on a global scale 1. The mainstream therapeutic modalities for OSCC have historically relied upon surgery followed by radiation or chemoradiation thus far. Furthermore, a targeted therapy that antagonizes the downstream signaling of the epidermal growth factor receptor (EGFR) has been proved to have synergy with radiotherapy in the treatment of mouth cancer 2. Lately, a series of specific antibodies directed against immune checkpoint molecules have exhibited a prolonged survival and improved prognosis in OSCC, in particular combined with chemo- or radio-therapy 3. Even though these therapeutic strategies are implemented, survival rates of OSCC patients have not been considerably enhanced (roughly 50%) 4, mainly as a result of cancer recurrence and metastasis 5, 6. This necessitates the exploration of complementary treatment options capable of tackling these issues. In such framework, the discovery of naturally-sourced and bioactive agents displaying a limited adverse effect for the management of OSCC has come to the fore as a key domain of research.

Sulforaphane (SFN), belonging to a class of plant-derived isothiocyanates, was shown to deliver various biological benefits for cancer prevention 7. Sulforaphane-cysteine (SFN-Cys), as one of naturally-occurring SFN metabolites, is more abundant in plasma and numerous organs (e.g. lung and kidney) and exhibits a longer half-life *in vivo*, in comparison with SFN 8. To data, SFN-Cys has been demonstrated to exert a wide range of anti-cancer properties in different types of tumors 9-13. In prostate cancer, SFN-Cys caused disruption of microtubule structures to trigger apoptotic responses 9 and downregulated the expression of galectin-1 to suppress cell invasion 10. Due to its potential to penetrate the brain-blood barrier, SFN-Cys was shown to impede cell cycle progression 11, and induce apoptosis in human glioblastoma (GBM) cells 12. Moreover, another study on GBM migration and invasion revealed that the mitophagy pathway, characterized by the removal of damaged mitochondria through autophagy, was inhibited by SFN-Cys to suppress the invasiveness of GBM cells 13. Notably, co-treatment with paclitaxel and SFN-Cys resensitized paclitaxel-resistant non-small cell lung cancer cells to paclitaxel through a caspase 3-dependent degradation of α-tubulin 14. Even though these *in vitro* and *in vivo* evidence has indicated anti-cancer features of this SFN metabolite, oncostatic effects of SFN-Cys on OSCC remain mostly elusive. Here, we attempted to clarify whether SFN-Cys interferes with oral tumorigenesis and further investigated the underlying molecular mechanisms. Our findings provided possible avenues for the application of a natural compound in the treatment of OSCC.

## Materials and methods

### Cell lines and chemicals

SCC-9 is derived from a human tongue squamous cell carcinoma, and HSC-3, also originating from tongue carcinoma, is known for its highly aggressive behavior. SCC-9 and HSC-3 were purchased from the American Type Culture Collection (Manassas, VA, USA) and maintained in MEM medium (Life Technologies, Grand Island, NY, USA) supplemented with 10% fetal bovine serum (FBS) (Gibco, Grand Island, NY, USA) as described previously 15, 16. D, L-Sulforaphane-L-cysteine (SFN-Cys) was commercially acquired from Santa Cruz Biotechnology (Dallas, Texas, USA). U0126 and SB203580 were purchased from Sigma-Aldrich (St. Louis, MI, USA), and JNK-IN-8 was obtained from Calbiochem (San Diego, CA, USA).

### Cytotoxic evaluation of SFN-Cys

The extent to which SFN-Cys is cytotoxic to OSCC cells was assessed with a microculture tetrazolium (MTT) colorimetric assay (Sigma-Aldrich) as previously described 17. SCC-9 and HSC-3, were cultured with different concentrations of SFN-Cys for 24 hr and then evaluated for cell viability per the production of formazan after dissolution in isopropanol, which was measured at 563 nm with a spectrophotometer (DU640, Beckman Instruments, Fullerton, CA).

### Flow cytometry

For determining the phase of cell cycle, the amount of DNA was measured via flow cytometry as previously described 18. In brief, cells in response to different concentrations of SFN-Cys for 24 hr were labeled with propidium iodide (PI) (Invitrogen, Carlsbad, CA, USA) and monitored with a BD AccuriTM C6 Plus personal flow cytometer (BD Biosciences, San Jose, CA, USA). For evaluating the progression of cell apoptosis, phosphatidylserine flipping from the inner to the outer leaflet of the plasma membrane was determined by using an FITC-labeled Annexin-V/PI Apoptosis Detection kit (BD Biosciences, San Jose, CA, USA) as stated previously 19. OSCC cells treated with SFN-Cys for 24 hr were harvested, followed by labeling of phosphatidylserine and DNA with FITC-labeled Annexin-V and PI, respectively, for 20 min. The proportion of cells positive for Annexin-V or PI was assessed by using flow cytometry.

### Apoptosis-related proteome analysis

For profiling of apoptosis-related proteins in SFN-Cys-treated OSCC cells, a Proteome Profiler Human Apoptosis Array Kit (R&D Systems, Minneapolis, MN, USA) that allows to detect 35 human apoptosis-related proteins simultaneously was used 20. Lysates of OSCC cells treated with and without SFN-Cys were collected, and 200 μg of total protein was subjected to each array per manufacturer's instructions. Pixel density of apoptotic protein markers was measured and normalized to the signal of reference array spots to calculate the levels of their relative expression.

### Western blotting

OSCC Cells with or without a 2-hr pretreatment of a kinase inhibitor for 2 hr were under exposure to SFN-Cys for 24 hr, washed, and collected in ice-cold lysis buffer. 20 μg of total protein was analyzed with SDS-PAGE assays 21. Detection of each cell marker was through hybridization with a number of specific immunoglobulins. Visualization was conducted with HRP-conjugated secondary antibodies (Dako Corporation, Carpinteria, CA, USA). Densitometry of immunoblots was analyzed through the ImageJ software.

### Statistical analysis

Data represent mean ± standard deviation (SD) of at least three separate experiments unless otherwise indicated. Statistical significance is based on a *p* value of <0.05 by employing Student's *t*-test.

## Results

### SFN-Cys is an effective inducer of cell death in OSCC

**Figure 1:**
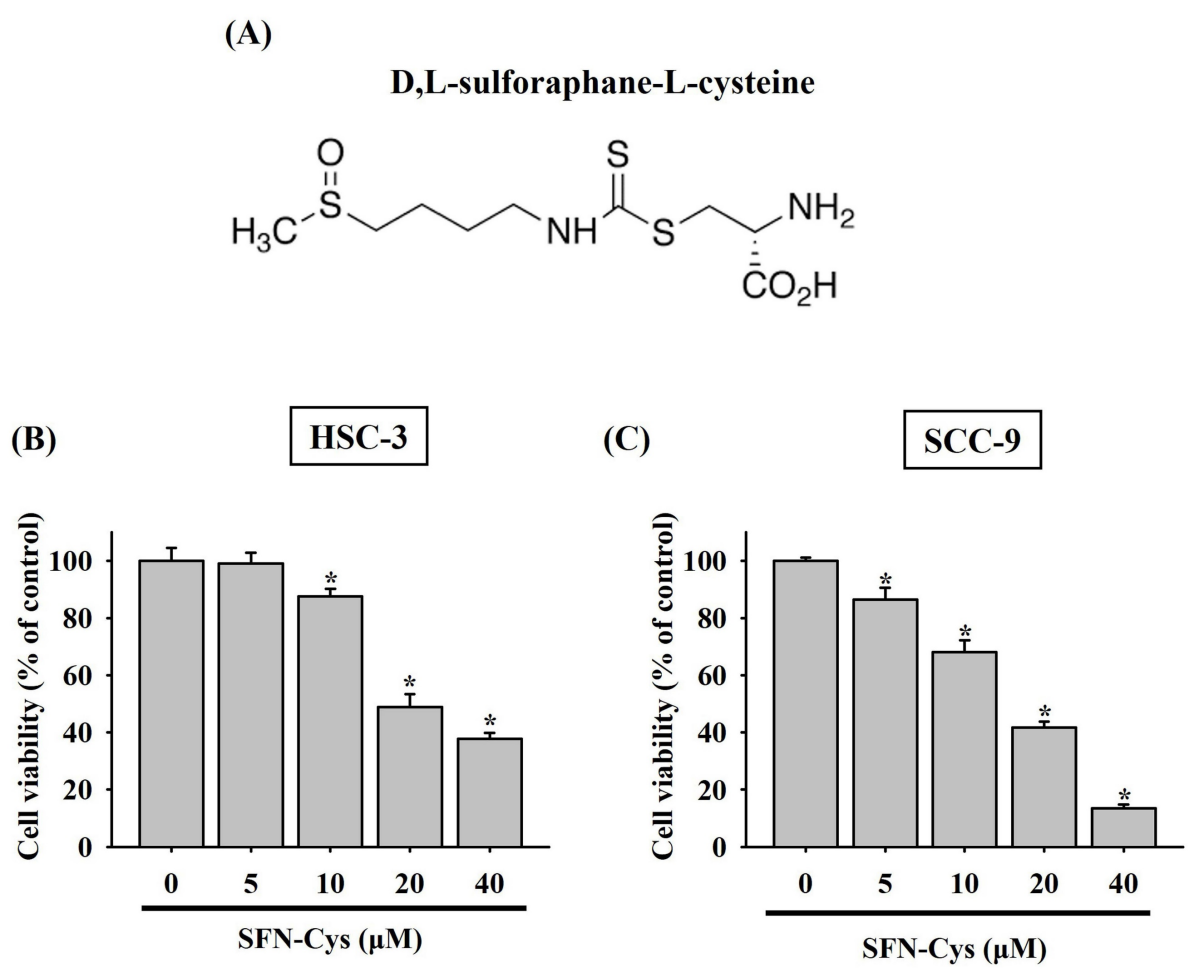
** Cytotoxic effect of SFN-Cys on OSCC cells. (A)** Structural formula of sulforaphane-cysteine (SFN-Cys). **(B-C)** SFN-Cys is cytotoxic to OSCC cells. HSC-3 **(B)** and SCC-9 cells **(C)** were treated with SFN-Cys at various concentrations for 24 hr and evaluated for cell viability. Data represent mean ± SD from three independent experiments. *p < 0.05, compared with untreated controls using Students t-test.

To clarify whether there is a tumor-suppressive effect of SFN-Cys on oral tumorigenesis, the cytotoxicity of SFN-Cys at different concentrations (5 to 40 μM) was tested in two OSCC cell lines, HSC-3 and SCC-9. In accordance with its anti-cancer features noted in other cancer types 9, 11, 14, our results demonstrated that addition of SFN-Cys reduced the viability of both HSC-3 and SCC-9 cells in a dose-dependent manner (**Figure 1**), suggesting an oncostatic role of SFN-Cys in OSCC.

### SFN-Cys stimulates cell cycle arrest and apoptotic responses in OSCC

**Figure 2:**
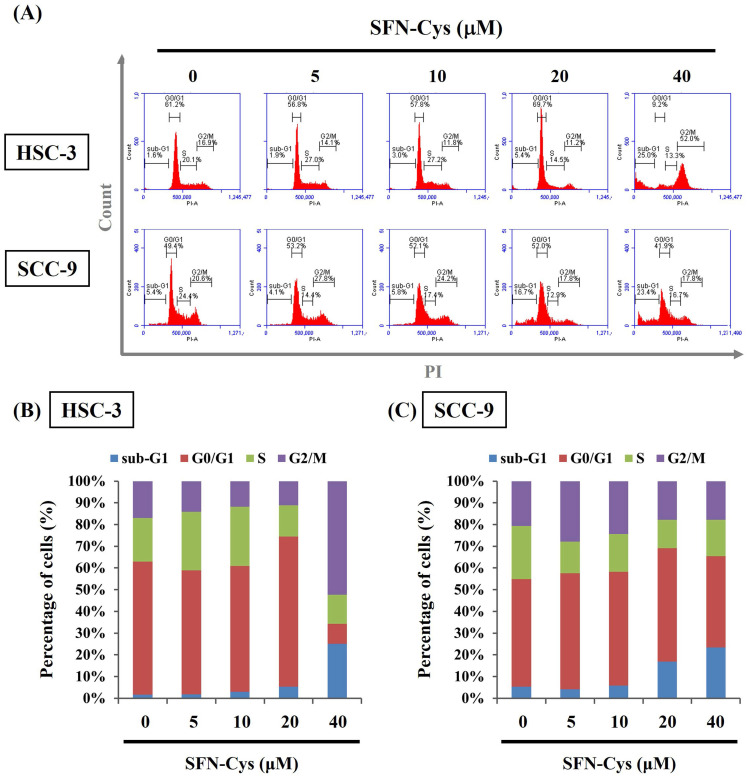
** SFN-Cys induced cell cycle arrest at sub-G1 phase in OSCC. (A)** Two cell lines were incubated with SFN-Cys at indicated concentrations for 24 hr and assessed for cell cycle distribution by monitoring DNA content via flow cytometry. **(B-C)** Quantification for proportion of SFN-Cys-treated HSC-3 **(B)** and SCC-9 cells **(C)** at individual phases of cell cycling.

**Figure 3:**
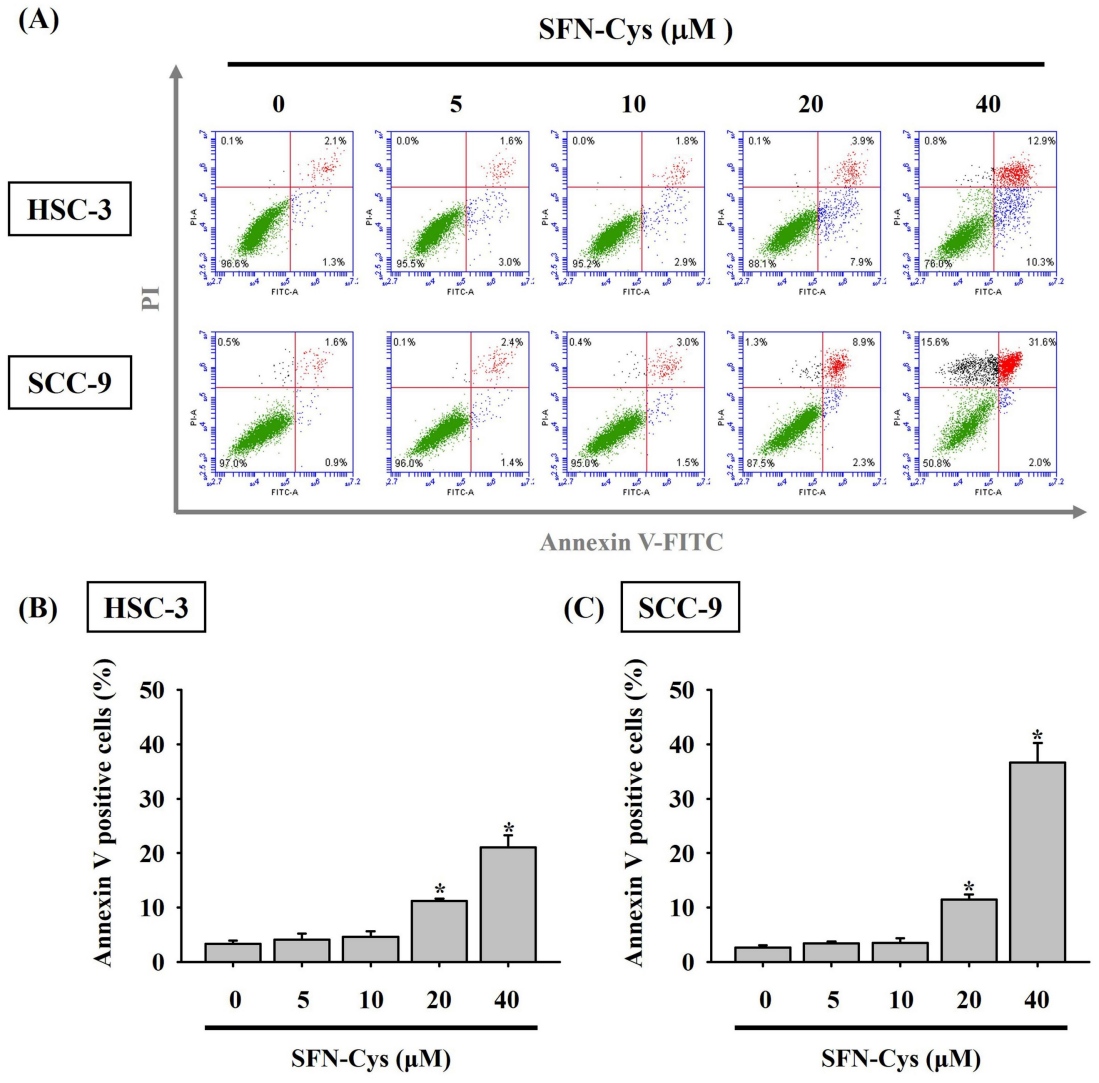
** SFN-Cys triggers cell apoptosis in OSCC. (A)** Two cell lines were under a 24-hr treatment with indicated concentrations of SFN-Cys, stained with PI and annexin-V, and examined for the status of cell apoptosis by flow cytometry. **(B-C)** Quantification for the proportion of cells undergoing apoptosis in SFN-Cys-treated HSC-3 **(B)** and SCC-9 cells **(C)** as determined by the ratio of cells positive for annexin-V staining. Data are shown as mean ± SD from three independent experiments. *p < 0.05, compared with untreated controls using Students t-test.

Given the finding that SFN-Cys effectively inhibited the growth of OSCC cells, we subsequently investigated whether such cytotoxic effect is related to the impediment of cell cycle or induction of cell apoptosis in OSCC. Our flow cytometry analysis of cellular DNA content revealed an increased proportion of both cell lines accumulated at the sub-G1 stage under the treatment of SFN-Cys (**Figure 2**), indicating its promotive effect on stalling cell cycle progression. It is plausible that such inhibition of cell cycling could initiate repair mechanisms or progress into apoptotic events in SFN-Cys-treated OSCC cells. By labeling of phosphatidylserine on the outer surface of cell membranes and DNA in cells with compromised membranes with Annexin-V and PI, we observed that treatment of both cell lines with 20 μM of SFN-Cys led to a significant elevation in the proportion of annexin-V-positive cells (**Figure 3**). This effect on promoting apoptosis was enhanced with the increase of SFN-Cys concentrations, indicating that the cytotoxicity of SFN-Cys in OSCC cells is accompanied with blockage of cell cycling and induction of programmed cell death.

### SFN-Cys treatment reshapes apoptosis-related proteome

**Figure 4:**
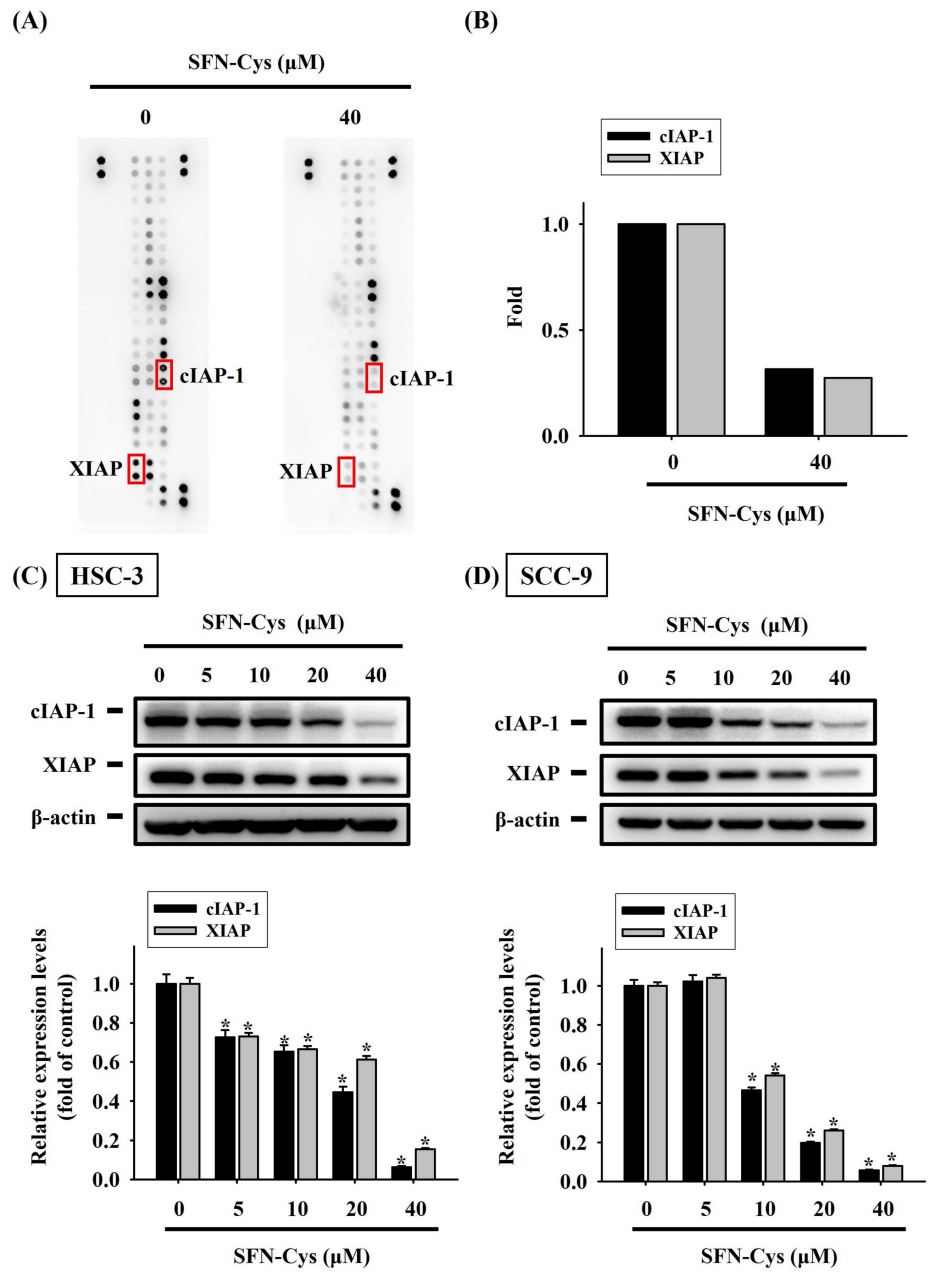
** SFN-Cys attuned an apoptosis-associated protein regulatory program in OSCC. (A)** Representative images of protein array membranes denoting expression levels of apoptosis-associated proteins in SFN-Cys-untreated and -treated HSC-3 cells. Spots of differentially expressed proteins are marked, labelled, and selected for further verification. **(B)** Quantification for pixel intensity of selected spots from HSC-3 samples **(C-D)** Validation of cIAP-1 and XIAP downregulation. HSC-3** (C)** and SCC-9 cells** (D)** were treated with SFN-Cys for 24 hr at various concentrations and tested for the expression of two apoptosis suppressors, cIAP-1 and XIAP, via Western blotting. Signals in each condition were quantified, normalized with internal controls (β-actin), and shown underneath. Data represent mean ± SD of three independent experiments. *p < 0.05, compared with untreated controls using Students t-test.

To better understand the underlying mechanism at the molecular level during SFN-Cys-induced apoptosis, we explored the proteome of SFN-Cys-treated HSC-3 cells related to cell apoptosis via simultaneously analyzing 35 apoptosis-associated proteins with a protein array. Fluctuations in the levels of many markers were detected in SFN-Cys-treated HSC-3 cells (**Figure 4A**). Of note, two suppressors of cell apoptosis, cellular inhibitor of apoptosis protein-1 (cIAP-1) and X-linked inhibitor of apoptosis protein (XIAP), were dramatically downregulated under SFN-Cys treatment (**Figure 4B**). The decline in the expression of cIAP-1 and XIAP by SFN-Cys was further validated as our Western blotting analysis revealed a suppressive effect of SFN-Cys on the levels of two markers in both HSC-3 and SCC-9 cells (**Figure 4C-D**), suggesting that addition of SFN-Cys attunes the apoptosis-associated proteome in OSCC.

**Figure 5:**
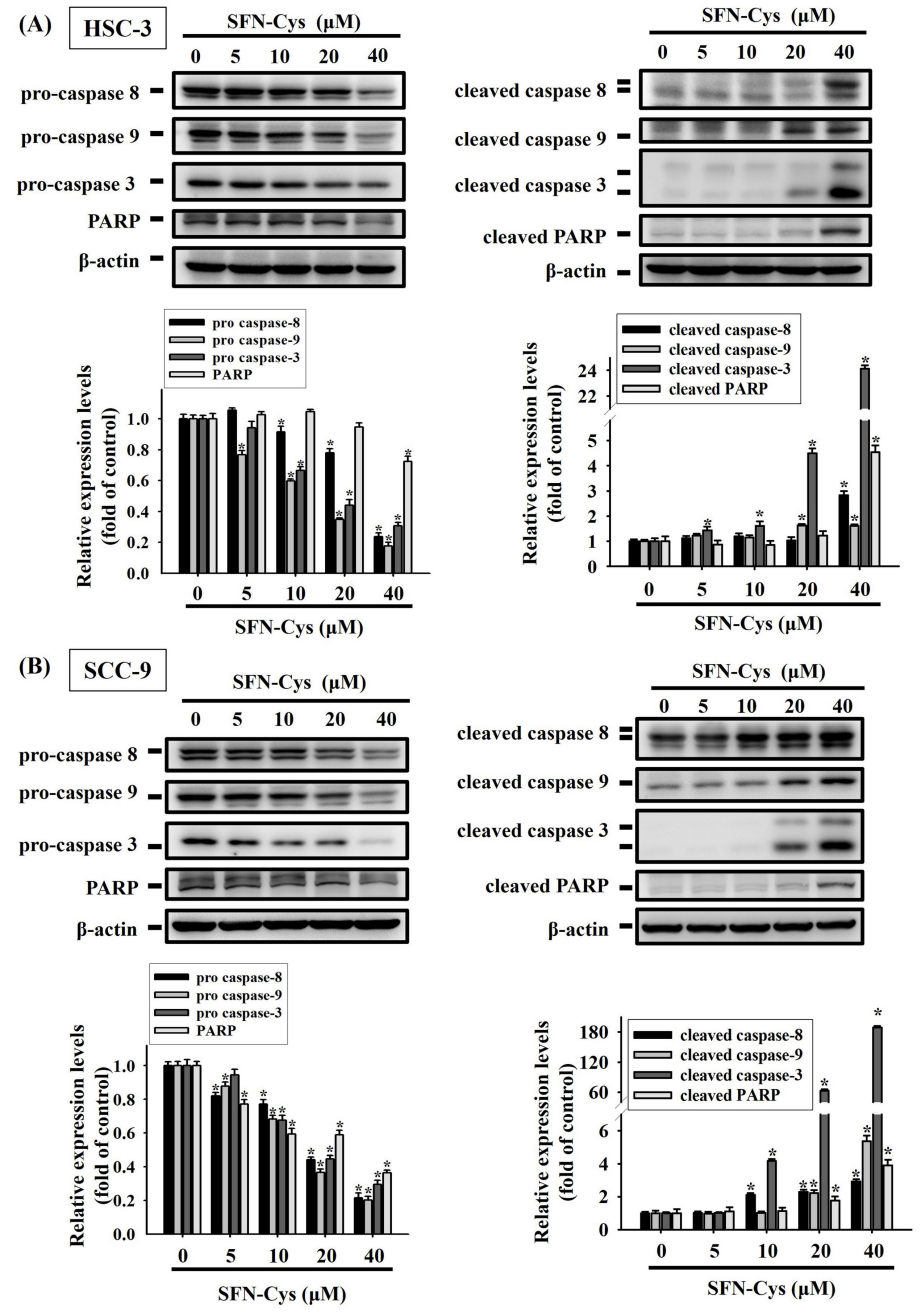
** SFN-Cys promotes the cleavage of pro-caspases and PARP in OSCC.** HSC-3** (A)** and SCC-9 cells** (B)** were exposed to various concentrations of SFN-Cys for 24 hr and examined for the levels of precursor or intact (left panel) and cleaved forms (right panel) of caspases and PARP via Western blotting. Comparisons of expression levels were shown in the right. Data are mean ± SD of three separate experiments. *p < 0.05, compared with untreated controls using Students t-test.

Moreover, we examined how SFN-Cys affects other molecular hallmarks of apoptosis. Our results showed that exposure to SFN-Cys reduced the levels of precursor or intact forms of caspase-3, -8, -9 and poly (ADP-ribose) polymerase-1 (PARP) (**Figure 5**). This response was in accordance with the observation that SFN-Cys induced the proteolytic cleavage of these apoptosis makers in two cell lines. Collectively, these data unveil an apoptosis-associated protein regulatory program in SFN-Cys-treated OSCC, underlined by downregulated apoptosis suppressors and activated caspase cascades.

### MAPK activation in SFN-Cys-treated OSCC

**Figure 6:**
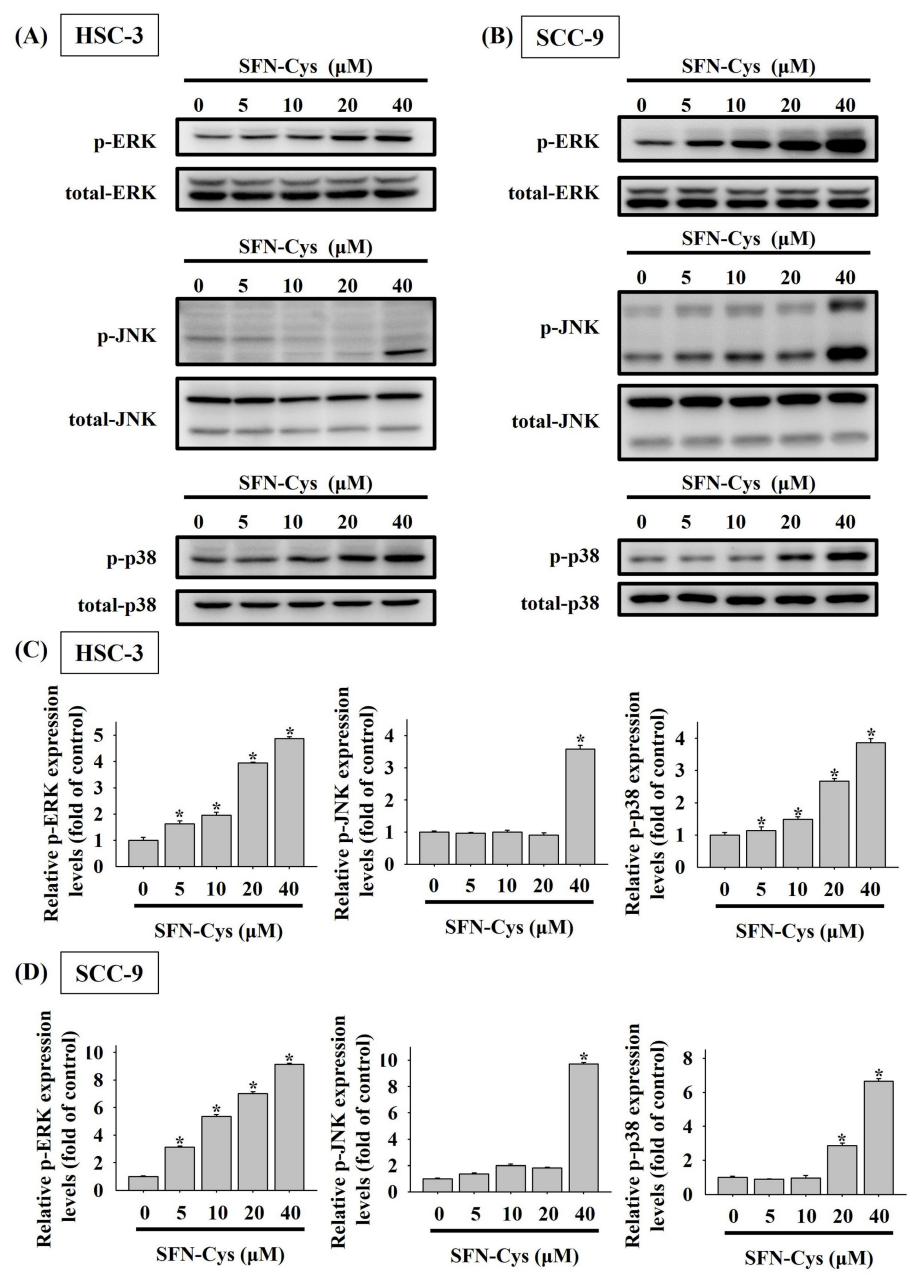
** SFN-Cys activates MAPKs in OSCC cells.** HSC-3** (A)** and SCC-9 cells** (B)** were treated with various concentrations of SFN-Cys and assessed for the levels of phosphorylated ERK1/2 (ERK), JNK, and p38 kinase via Western blotting. Relative expression levels of phosphorylated forms in each condition of HSC-3** (C)** and SCC-9 cells **(D)** were quantified and normalized with total kinase levels. The values represent mean ± SD of three independent experiments. *p < 0.05, compared with untreated controls using Students t-test.

Mitogen-activated protein kinases (MAPKs) play a crucial role in regulating programmed cell death, either promoting or inhibiting it depending on the nature of external stimuli 22-24. Accordingly, we investigated SFN-Cys-mediated activation of MAPKs in OSCC and found distinct activation patterns among ERK, JNK, and p38 kinase (**Figures 6A-D**). Specifically, a dose-dependent effect of SFN-Cys on activation of ERK and p38 was seen in both cell lines, whereas profound phosphorylation of JNK was only detected in HSC-3 and SCC-9 cells exposed to SFN-Cys at 40 μM, suggesting a differential regulation of MAPKs by SFN-Cys in OSCC cells.

### Functional involvement of JNK in SFN-Cys-stimulated caspase activations

**Figure 7:**
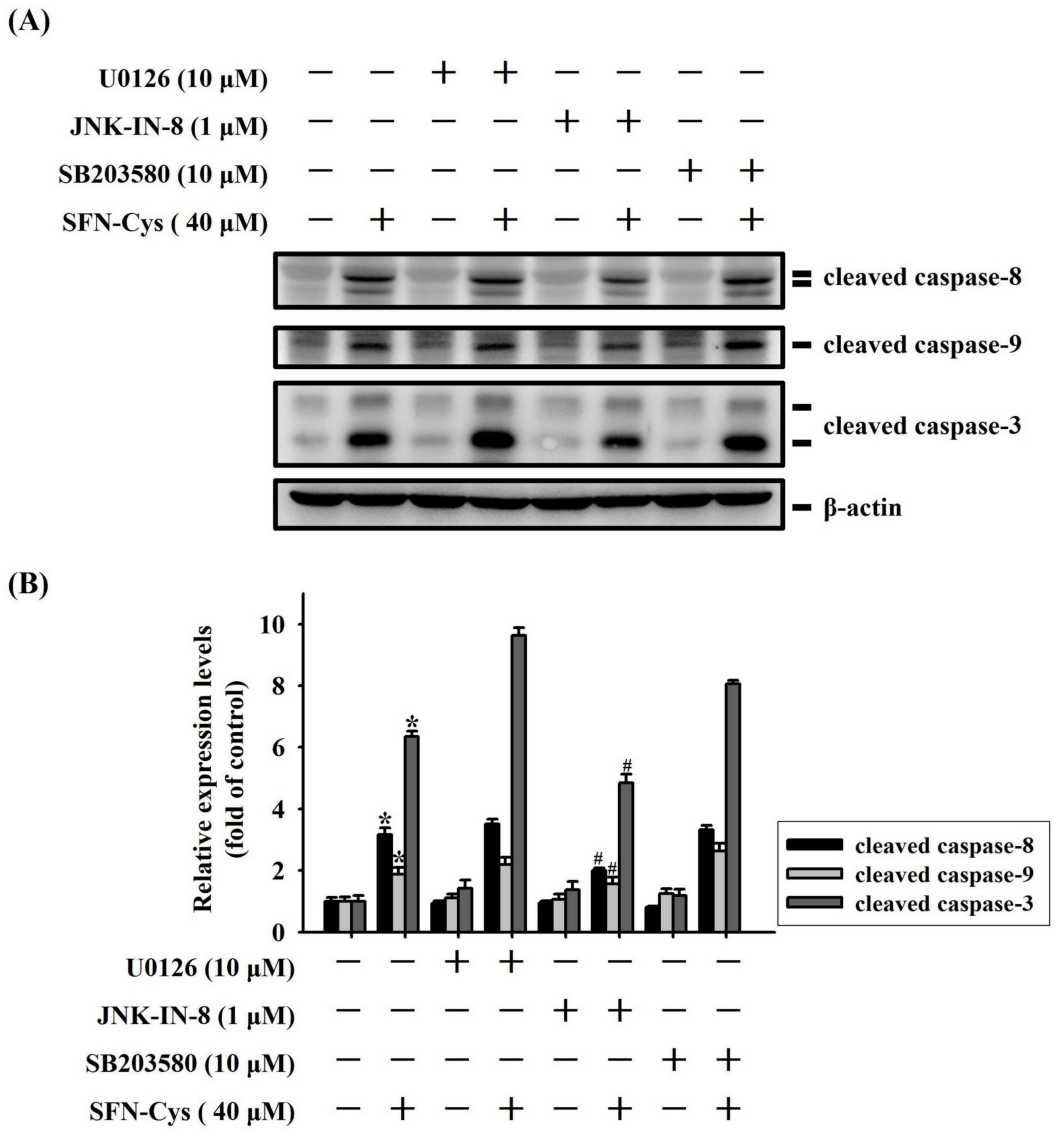
** JNK signaling is implicated in caspase activation of SFN-Cys-treated OSCC. (A)** HSC-3 cells were pre-incubated with individual kinase antagonist for 2hr and subsequently treated with SFN-Cys for another 24 hr, followed by assessment for the levels of caspase cleavage. **(B)** Signal intensities for cleaved caspases in SFN-Cys-treated HSC-3 cells were quantified and normalized to levels of internal controls (β-actin). Data represent average ± SD of three independent experiments. *p < 0.05, compared with untreated controls using Students t-test. #*p <* 0.05, compared with SFN-Cys-treated cells using Students t-test.

We next explored whether MAPK activity is functionally connected to caspase cascades triggered during the apoptotic events in SFN-Cys-treated OSCC. Our analyses demonstrated that SFN-Cys-induced cleavage of pro-caspase-8, -9, and -3 in HSC-3 cells was interfered with the addition of a specific JNK blocker (JNK-IN-8) but not affected by pretreatment with an ERK or p38 antagonist (**Figure 7**). This result links the activity of JNK kinase to caspase activation in SFN-Cys-treated OSCC cells.

## Discussion

Poor prognosis and survival of patients with advanced forms of oral cancer still post a considerable challenge, as encouraging outcomes have been obtained in early-stage OSCC patients who received contemporary treatment strategies. Therefore, there is a necessity to implement complementary therapeutic options to alleviate the burden of illness. It is well documented that naturally-occurring compounds derived from botanicals exert a synergistic effect on cancer management when used in combination with standard treatment methods 25. Here, we exhibited that SFN-Cys, a natural metabolite of plant-derived isothiocyanates, elicited an inhibitory effect on the viability of OSCC cells, concomitant with elevation of cell apoptosis. Further elucidation of SFN-Cys's actions at the molecular level revealed a downregulation of several apoptosis suppressors and a JNK-mediated activation of caspase pathways. Our findings suggest an indication for the use of this natural substance in OSCC treatment.

Cumulative experimental findings have exhibited that SFN and its derivatives give rise to selective interference with progression of various malignancies 26 through dose-specific mechanisms and regulation of different molecules. SFN-Cys is one of SFN derivatives that has been shown to be more effective than the parent compound due to its high bioavailability 8. Exposure of lung cancer cells to SFN-Cys caused abnormal cell morphology, which was coupled with microtubule disruption and leading to apoptosis 27, implying SFN-Cys as a potent microtubule-interfering agent. Inhibition of microtubule dynamics induces cell cycle arrest and the release of signaling molecules that regulate apoptotic responses, such as Bim and survivin 28. Consistently, blockage of cell cycling was observed in our study where SFN-Cys interfered with the proliferation of OSCC cells. In addition to cytoskeletal proteins, SFN-Cys treatment downregulated CDK-4 and -6, leading to programed cell death in human GBM cells 11. The primary mechanism of actions for CDK-4 and -6 is suppression of Retinoblastoma (RB) protein phosphorylation and thus inhibition of the G1/S transition 29, in accordance with our observation that both SFN-Cys-treated SCC-9 and HSC-3 cells were accumulated at the sub-G1 stage. Furthermore, the tumor-suppressive effect of SFN-Cys on activating caspases and promoting apoptosis was mediated by ERK1/2 signaling in GBM 30 and prostate cancer cells 9. However, pretreatment of OSCC cells with U0126 failed to suppress SFN-Cys-stimulated cleavage of caspases in HSC-3 cells. Instead, we observed that SFN-Cys-induced OSCC apoptosis involved a JNK-dependent activation of caspase cascades. JNK pathway has been functional linked to the intrinsic apoptotic pathway transduced from the mitochondria as well as the extrinsic apoptotic events initiated by death receptors 31. In sepsis-associated central nervous system damages, SFN protected microglial cells from lipopolysaccharide-induced inflammation by inhibition of JNK signaling 32. Our data, joined with others' findings, suggest that SFN-Cys is an effective inducer of programed cell death in OSCC, employing a JNK-mediated activation of caspase cascades.

Along with promotion of caspase activation, we demonstrated a downregulation of several apoptosis suppressors in SFN-Cys-treated OSCC. Among these SFN-Cys-regulated proteins, cIAP-1 and XIAP both function as E3 ubiquitin ligases that mediate NF-*κ*B signaling and apoptotic responses through the ubiquitylation of key constituents of TNF and Fas receptor complexes and 33, 34. In prostate cancer cells exposed to SFN treatment, a marked decrease in the levels of inhibitor of apoptosis (IAP) family proteins (cIAP-1, cIAP-2 and XIAP) was detected, which was accompanied by inhibition of NF-*κ*B activation 35. However, the levels of XIAP, cIAP-1 and cIAP-2, remained unchanged in the SFN-treated cervical carcinoma cells, as SFN substantially inhibited the levels of cIAP-1 expression in liver cancer cells without affecting the XIAP and cIAP-2 expression 36, although SFN efficiently triggered apoptosis in both malignant cell lines. Mammalian XIAP and cIAP-1 share several functional features, including the ability to bind caspases, the ability of self-ubiquitination and ubiquitination, and neddylation of caspases via their RING domain 37. During the process of oral carcinogenesis, it was found that oral tissues that underwent malignant transformation displayed high levels of IAP proteins, as normal buccal mucosa exhibited DNA hypermethylation but did not express IAP proteins 38. These suggest that downregulation of cIAP-1 and XIAP facilitates programmed cell death and sensitizes OSCC cells to extracellular apoptotic inducers.

In this study, an oncostatic potential of SFN-Cys on oral tumorigenesis via blockage of cell cycling and promotion of apoptotic events was reported. However, extra efforts are needed to address several study limitations. One issue is that, despite our *in vitro* evidence showing an anti-cancer effect of SFN-Cys on OSCC, the influence of this natural SFN metabolite could be incompatible due to distinctive tissue distribution profiles *in vivo*. As the differences in bioavailability and distribution of specific SFN metabolites to tissues were noted 8, the extent of which SFN metabolites reach target tissues of carcinogenesis remains a concern. Further investigations using additional rodent models will be beneficial for validating the tumor-suppressive characteristics of SFN-Cys during oral tumorigenesis. Another caveat is that two cell lines tested in our experiments were initially isolated from the tongue of OSCC patients. It is found that different mutational signatures, etiology, and survival rate are associated with malignancies developed from different anatomical regions of oral cavity (for instance, tongue, lip, buccal mucosa, and gingiva) 39. Exploration of SFN-Cys's actions with additional OSCC cell models derived from other anatomical sites is warranted to reinforce the generalizability of major findings.

In conclusion, we showed that SFN-Cys elicited programmed cell death and cell cycle arrest in OSCC cell lines, involving activation of caspase pathways via the JNK signaling. Our findings offer possible avenues for the application of a natural compound in fighting against OSCC.
